# Fracturing evolution of red sandstone: insights from three-point bending experiment and numerical simulation considering material inhomogeneity and internal discontinuities

**DOI:** 10.1007/s10064-025-04204-3

**Published:** 2025-03-11

**Authors:** Xiangxin Liu, Bin Gong, Zhengzhao Liang, Zhengnan Zhang, Xun You

**Affiliations:** 1 School of Civil and Surveying & Mapping Engineering, Jiangxi University of Science and Technology, Ganzhou, 341000 China; 2 Department of Civil and Environmental Engineering, Brunel University of London, London, UB8 3PH UK; 3 State Key Laboratory of Coastal & Offshore Engineering, Dalian University of Technology, Dalian, 116024 China; 4 School of Resource and Environmental Engineering, Jiangxi University of Science and Technology, Ganzhou, 341000 China

**Keywords:** Three-point bending, Rock fracturing, Acoustic emission, Infrared radiation, Internal discontinuities, Numerical modeling

## Abstract

To reveal the various propagation paths of micro-cracks under the continuous process of stress buildup, stress shadow, and stress transfer, three-point bending experiments and numerical simulations were carried out by considering material inhomogeneity and internal discontinuities. The characteristics of red sandstone fracturing evolution were analyzed from the aspects of acoustic emission (AE) energy index, infrared radiation (IR) changes, fracture surface roughness, stress fields and so on. The test results indicate that four stages are divided in the gradual process of energy release of red sandstone fracturing under three-point bending test, the rough fracture surfaces of crack were extremely small, tensile crack makes the largest proportion. IR and AE perform some significant precursor information of rock fracturing, *e.g.*, a large amount of high-temperature debris scattered in infrared thermography, the maximum value of AE accumulative energy and the concentration effect of AE events location. Different tensile stress level has different features, macroscopic fracture morphology happens in a low level, and micro-cracks appears in the weakness of crystal surfaces in a high level. It needs to be emphasized that five different modes, pass through, crack-tip blunting, extended-back, crack-forking and passing round, were concluded in terms of the repeated process of stress buildup, stress shadow & stress transfer. These achievements contribute to the better understanding of the failure mechanisms of red sandstone.

## Introduction

Red sandstone is a typical non-heterogeneous and discontinuous rock materials, its fracturing process often shows the coexistence of tension-shear. The development of internal primary cracks reflects the main process of rock fracturing evolution (Clayton 2010; Chen et al. 2022a, b; Yu et al. 2024; Tang et al. 2025). Tensile failure is a key factor in the fracturing evolution of red sandstone. Quantitative indicators of red sandstone tensile failure, such as direct tensile strength and fracture toughness, are crucial properties for engineering construction (Wu et al. 2016; Knippel et al. 2023; Yang et al. 2023; Cao et al. 2025). Three-point bending test provides a proven approach to understand the rock tensile fracturing behavior. Some researchers have investigated the tensile characteristics using three-point bending tests under different conditions (Exadaktylos et al. 2001a, 2001b; Brune et al. 2013; Kuruppu et al. 2014). In terms of fracture mechanisms, the rock samples with various single notches were tested to investigate their fracture behaviors, and the modified criterion and three-parameter model (based on *K*_*I*_, *K*_*II*_ and *T*) were developed to calculate the crack-tip stresses (Lim et al. 1993; Ayatollahi and Aliha 2007; Aliha et al. 2013; Maina and Konietzky 2024; Zhang et al. 2024a). The stiffness of a pre-cracked three-point bend sample was also calculated using the shear deformation and the rotary inertia through the vibration analysis method (Jiang et al. 2004). Furthermore, the simplified method to determine the double-*K* fracture factors, *i.e.*, ${K}_{Ic}^{\text{ini}}$ and ${K}_{Ic}^{\text{un}}$, through three-point bending test was proposed, and two empirical formulae were established and proven to be accurate for a large range of *a/D* (Xu and Reinhardt 2000). Besides, the sample diameter as well as crack type show great effect on the monitored fracture toughness. But the load rate, crack size & sample thickness may produce negligible influence on the fracture toughness (Khan and Al-Shayea 2000; Skarżyński and Tejchman 2010; Li et al. 2023). Although some research has been conducted, the influence of the continuous stress processes—encompassing stress buildup, stress shadow, and stress transfer—on the diverse propagation paths of micro-cracks influenced by the internal discontinuities of red sandstone remains unclear.

Simultaneously, effective monitoring is essential to capture the fracturing process during the evolution of rock failure. Acoustic emission (AE) monitoring has become increasingly prevalent in the field of rock failure analysis (Mogi 1962; Labuz et al. 2001). The AE events occur because the elastic strain energy releases as the elastic waves when rock gets fractured. Through monitoring AE events, rock fracturing is analyzed quantitatively in terms of the spatiotemporal characteristics of elastic wave including generation time, frequency, amplitude, magnitude, etc. Furthermore, AE timing parameters are used to predict the crack development and to quantify the physical parameters of rock fracturing (Sagar et al. 2010; Ohno et al. 2014; Li and Einstein 2017; Giuseppe et al. 2018). A position of an AE event is also a valuable parameter reflecting spatial position, orientation & dip angle of crack growth (Ullah et al. 2023; Jiang et al. 2024; Zhang et al. 2025). With the load increasing, the distribution of AEs reflects the gradual process from the creation, development & penetration of micro-cracks to the generation of macro-fractures (Kuksenko et al. 2007; Song et al. 2023). Meanwhile, a direct source location algorithm was developed by selecting high-quality AE signals and incorporating waveform identification. This approach significantly enhanced the reliability and accuracy of AE source localization (Chen et al. 2017). In addition, Wu et al. (2000) and Huang et al. (2021) have applied the infrared radiation temperature (IRT) monitoring to explore rock fracture and related abnormal phenomenon. Liu et al. (2006) mentioned that the IRT variation could be greatly affected by failure modes. Yang et al. (2021) suggested that the IRT enhancement represented crack creation, development, and coalescence with rock fracturing. AE monitoring and IR monitoring were applied to capture the development of spatial cracks during three-point bending test in this paper. Liu et al. (2018) investigated the influence of water contents on rockburst in tunnels under dry and saturated granite rocks by the acoustic emission (AE) monitoring system and infrared thermal imager monitoring system. The results indicated that water conditions enhance the effects of infrared radiation temperature. Sudden temperature changes in the tunnel walls were used as a precursor to rockburst events on the spatial scale.

In this study, the AE monitoring technology, infrared thermography, scanning electron microscopy, and numerical simulation techniques were employed to analyze the fracture characteristics of red sandstone under three-point bending. AE signals were recorded to reveal the temporal and spatial evolution of micro-cracks, while microfracture morphology and surface roughness were examined to gain insights into the mechanisms of crack initiation and growth. Meanwhile, the mesoscopic simulation was conducted to reproduce the influence of the process of stress buildup, stress shadow, and stress transfer on the continuous development of cracks and explore potential fracture propagation paths affected by coarse particles.

## Three-point bending test

### Rock specimen preparation

**Fig. 1:**
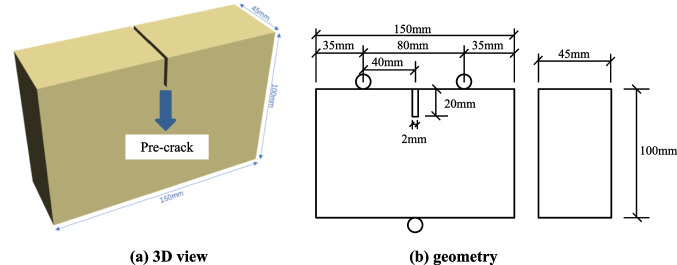
Shape and size of the rock specimen. (**a**) 3D view. (**b**) geometry

Red sandstone has been selected as the rock specimen, the size is set as 150 mm (length) × 100 mm (width) × 45 mm (height). The cuboids rock specimens have a pre-crack at the middle of the upper boundary with the size of 2 mm (width) × 20 mm (length) × 45 mm (thickness) (Fig. 1).

Specimens were tested according to the standard test procedure of engineering rock masses determined by GB/T50266-99 (National Standards of the People's Republic of China 1999). Clearly, the allowable deviation of unevenness on two ends was less than 0.05 mm, and the end faces were perpendicular to the axis of specimens, with a tolerance of ± 0.25°. Simultaneously, all specimens were cleaned and kept in a drying oven for 48 h subjected to the temperature of 105℃.

### Testing equipment and its setting

**Fig. 2:**
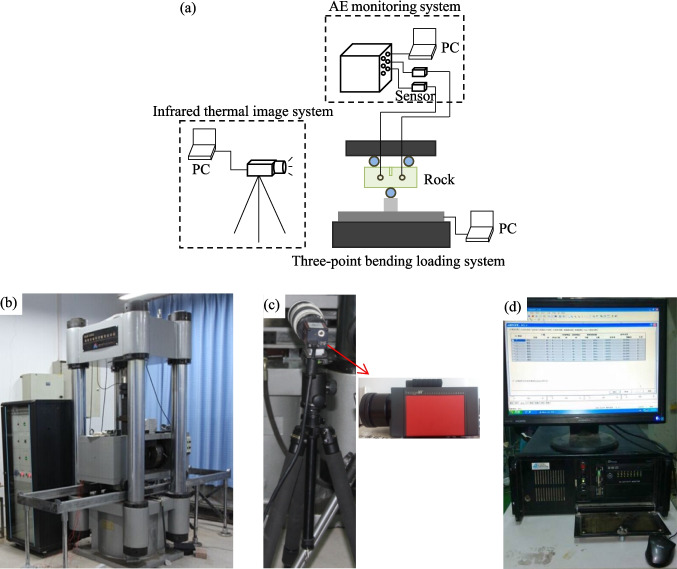
Test equipment including (**a**) composition of the test apparatus, (**b**) rock load system, (**c**) infrared thermal image system and (**d**) AE monitoring system

**Fig. 3:**
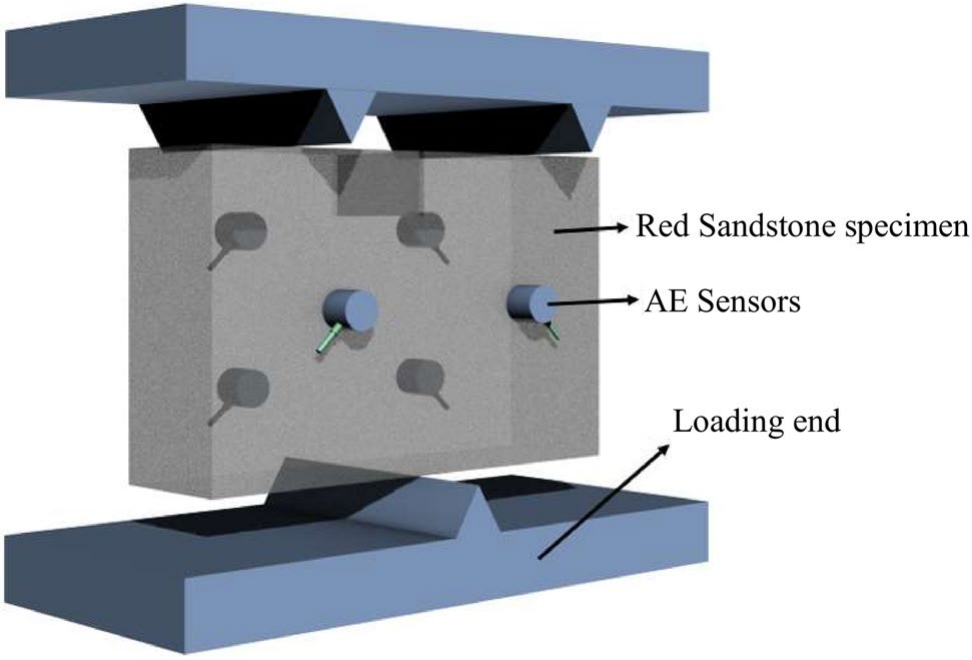
The load mold and AE sensor arrangement

Testing equipments are comprised of the rock testing system, AE monitoring system and infrared thermography system, as shown in Fig. 2. The advanced test equipment is to ensure the correctness of test data. TAW-3000 servo-rigidity test machine with the maximum axial load of 3000kN was adopted and the load error was less than 1%. After the rock sample was placed at the center of the bearing plate, the pre-load was conducted to ensure that the rock surface and the load end were fully coupled to avoid the noise signals caused by contact and friction that would affect acoustic emission (AE) monitoring date. The force-control method with the load rate of 200 N/s was applied. The PCI-2 AE system that can effectively capture AE spatial positions and collect AE time–frequency information was adopted. Prior to the test, the pre-amplifier threshold of the AE monitor was set to be 40 dB and the sampling threshold was set to be 45 dB. Meanwhile, the sensors were arranged as shown in Fig. 3 and the sensor model used in the test was RS-2A. A coupling agent was applied between the rock and the AE sensors to minimize the attenuation and distortion of AE signals. Initially, all testing equipment was activated simultaneously to ensure the real-time recording of both mechanical and AE data.

### Test monitoring results

1. Macroscopic fracture morphology analysis

**Fig. 4:**
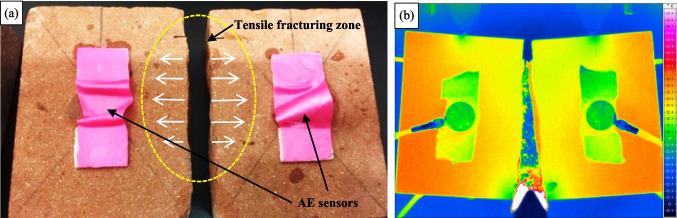
Rock macroscopic failure phenomena: (**a**) visible light and (**b**) infrared thermography

A tensile fracture zone occurred at the middle of the red sandstone sample, as shown in Fig. 4a. Firstly, some new cracks appeared at the tips of the existing cracks. Later, the newly-formed cracks developed downwards to the bottom with the load increasing. Finally, a through-type main fissure appeared, and the red sandstone specimen was divided into two pieces, as shown in Fig. 4b. The fracture morphology is relatively intact and undulatory, exhibiting typical brittle failure characteristics under low tensile stress conditions. The fracturing evolution of the rock specimen was simultaneously monitored using the infrared thermography system, as shown in Fig. 4b. A large amount of high-temperature debris scattered from the crack surface at the moment of macro failure in the rock. This phenomenon indicates that both tensile and shear fracturing occur under high tensile stress conditions, as also noted by Wu et al. (2006a and 2006b).

(2) Stage division during rock fracturing process

**Fig. 5:**
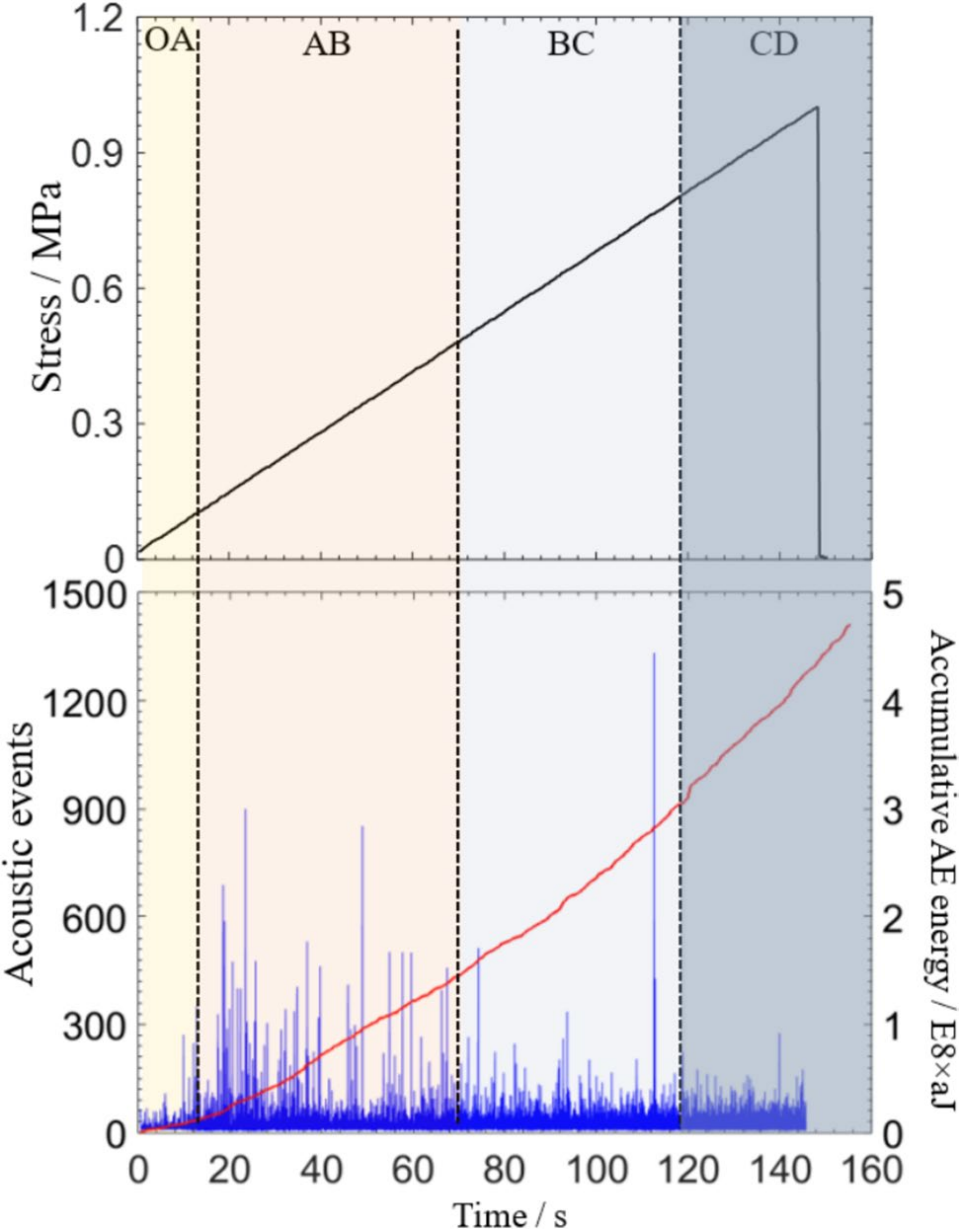
Evolution of AE events and accumulative AE energy during rock fracture

As the main form of strain energy release, AE events reflect the fracturing activities, and accumulative AE energy can manifest the sum of released energy through AE during the whole process of rock failure. Combined AE frequency and AE accumulative energy, the gradual evolution of energy release is effectively expressed, as shown in Fig. 5, four stages are distinguished as follows:

- **OA Stage**: At the beginning of load and AE activity is inactive. AE signals are generated through pre-existing crack compactions inside the rock. And the curves of AE events & accumulative AE energy are at the very low level.
- **AB Stage**: During stable crack expansion, AE events greatly grows up and becomes stable in the range of 200–300/s, and the accumulative AE energy performs as nearly linear growth. Micro-cracks inside the rock continuously develop at a steady rate.
- **BC Stage**: With the load increasing, the frequency of AE events slows down, but the accumulative AE energy keeps growing fast, indicating that large-scale cracks periodically occur inside the rock.
- **CD Stage**: AE count decreases sharply, and the accumulative AE energy rises minimally when the force reaches its peak. The stored energy inside the rock instantaneously releases, and the bearing capacity of the rock sample falls sharply before the occurrence of macro fracture.

Under the action of external force, natural grains, crystal planes, micro-fractures, voids and other defects lead to uneven local high-stress concentrations. At the beginning of loading, cracking speed slightly increases at the contact zone between the rock and the load end, but the cracked area is relatively small. AE events increase rapidly and remain active, and greatly change the accumulative energy of AEs. During the stage of unsteady crack expansion, crack propagating and interpenetrating clustering phenomenon occurs inside the specimen. The energy discharge rate significantly grows up. A special kind of AE waveforms are generated before final failure, and AE accumulative energy reaches its peak lastly.

(3) Crack mode analysis

The rise time/amplitude (RA) value is the ratio of the rise time to the amplitude, and the average frequency (AF) is determined as the ratio of the threshold crossing to the signal duration (Shiotani 2008; Ohtsu and Tomoda 2007). RA-AF scatter density contours (Zhang et al. 2024b) are used to determine crack mode which correspond to the morphological features of macroscopic fracturing. Namely, the RA-AF scatter density contour method is used to analyze fracture patterns and crack propagation in red sandstone by plotting RA against AF of acoustic emission signals on a scatter plot. The density of points on the scatter plot corresponds to the dominant fracture mechanisms (*e.g.*, tensile vs. shear fractures). Actually, tensile cracks are generally characterized by a short rise time and high amplitude. Namely, a relatively low RA value is related to the tensile performance of the fracture event. Simultaneously, AF is also a critical parameter used to determine crack mode. Its changing from a high value to a low value means a transition of the crack mode from stretching to shearing.

**Fig. 6:**
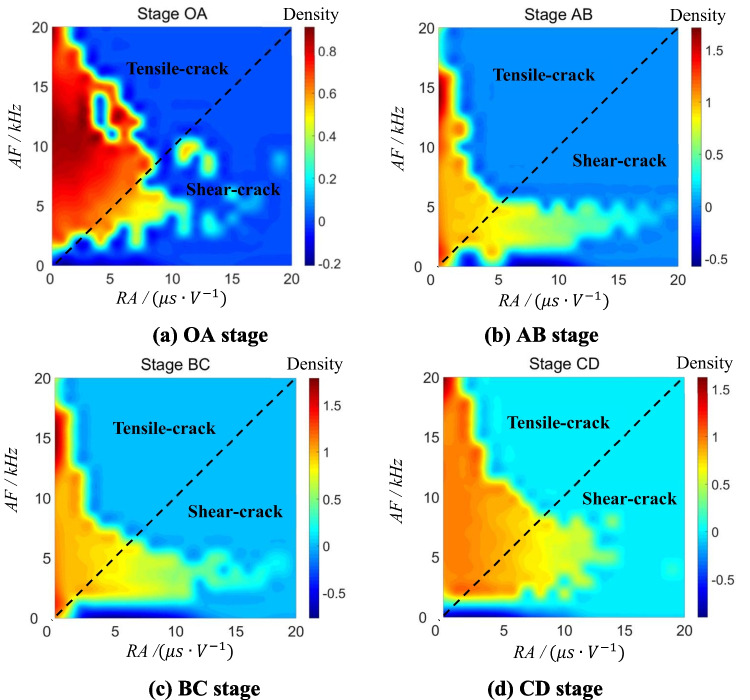
Density contours of RA-AF in each stage. (**a**) OA stage. (**b**) AB stage. (**c**) BC stage. (**d**) CD stage

Although the proportion of tensile cracks becomes greater than shear cracks during three-point bending test, different stages show different timeliness characteristics. In OA stage (Fig. 6a), tensile cracks occur more times than shear cracks. In AB (Fig. 6b) and BC stages (Fig. 6c), the frequency of shear cracks rises, but the proportion of tensile cracks is still greater than shear cracks. In CD stage (Fig. 6d), the proportion of shear cracks decreases until less than AB and BC stages, but keeps larger than OA stage. Therefore, the majority of cracks are tensile type combined with a small number of shear cracks. AE signals of rock fracture also indicate that most cracks are tensile type and only a small proportion of cracks are shear type during three-point bending test.

(4) Spatial distribution characteristics of AE events

**Fig. 7:**
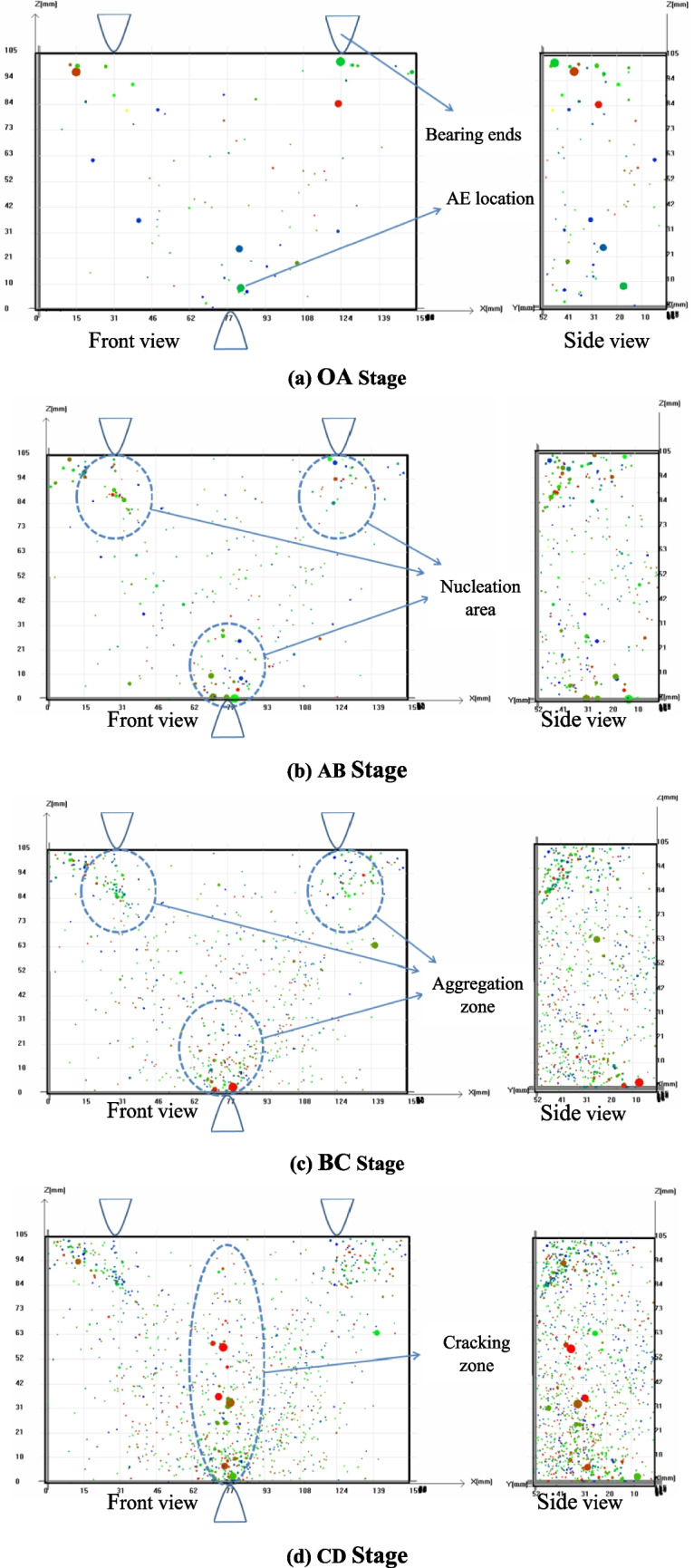
Spatial location of AE events during the rock fracturing evolution under three-point bending

Figure 7 shows the three-dimensional (3D) distribution of AE events located during rock fracturing evolution under three-point bending. The information of small colorful balls is used as to reveal the spatiotemporal development of AEs. Specifically, the different colors of the balls indicate the arrival time of the AE positioning point. For instance, the blue color denotes that the positioning point arrives first, and the red color denotes that positioning point arrives last. The radius of the ball is determined by the energy magnitude released by the AE event. The bigger the ball size is, the larger the energy value of the AE event is, and vice versa. The spatiotemporal evolution features of AE events during the rock fracturing in three-point bending test are expressed as follows:

- **OA Stage**: AE signals mainly originate from the micro-hole closure and micro-crack generation during the low-force load. Meanwhile, AE activity becomes more and more active and the number of anchor points gradually grows up. Although, balls distribution is relatively discrete, several large balls appear near the bearing and load ends.
- **AB Stage**: The internal positioning points gradually increase and gather in the vicinity of three bearing and load ends. Ball distribution generally evolves from a dispersed state to an initial formation of the crack nucleation zone near the load tip. A number of high-energy signals appear at the tips of the existing cracks, demonstrating that the strong and large-scale fracturing events shift toward the main fracture direction at the center of rock sample.
- **BC Stage**: AE points accumulate at the bearing/load ends and along main fracturing surfaces of rock with the load increasing. When the rock specimen becomes unstable, the creation, development, and coalescence of small and large cracks occur at the various scales inside the rock sample. Micro-cracks that assemble in the rock forms an aggregation region of AE events. The phenomenon of localized damage concentration emerges.
- **CD Stage**: Crack nucleation zone is basically built up, and a large number of high-energy event points appear in the vertical direction of the nucleation zone. The internal large-scale crack rapidly expands along the fracture surfaces, and the rock specimen is broken from the middle. During the near-destructive phase of rock, the internal damage gradually accumulates, and localized cracks are produced. Then, rock sample is destabilized and destroyed. A macroscopic tensile crack appears in the end.

Simultaneously, the spatial evolution of fracturing is represented by the scattered but regular AE events, and the locations of AE events intuitively reflects the internal fracturing behaviors, and visualized the process of crack creation, propagation and penetration. In early stage, AE events are random distribution, these energies are similarly small. As the loading goes on, AE events are located in the bearing ends and loading side, these energies are going to be larger and larger.

## Numerical simulations test

### Two-dimensional model

The rock failure process analysis (RFPA) method is developed based on the continuum mechanics, the damage mechanics & the statistical theory (Wang et al. 2023a; Liu et al. 2024; Gong et al. 2025a). The advantages of RFPA lies in modeling the failure process of rocks (Chen et al. 2022a, b; Wang et al. 2023b; Gong et al. 2024a, 2025b). To reflect the heterogeneous distribution of material properties, rock models are built up with numerous meso-elements within the RFPA. Meanwhile, the mechanical parameters of the elements vary from one another but obey a given distribution function. Weibull distribution function has been adopted widely and can be defined as follows (Gong et al. 2024b):

1 $$f\left(w\right)=\frac{m}{{w}_{0}}{(\frac{w}{{w}_{0}})}^{m-1}exp{(-\frac{w}{{w}_{0}})}^{m}$$

where ***w*** is the strength/elastic modulus of elements and ***w***_***0***_ is the mean value of the particular parameter ***w***. Besides, ***m*** is termed the heterogeneity index determining the shape of the probability density curve and represents the degree of material heterogeneity.

**Fig. 8:**
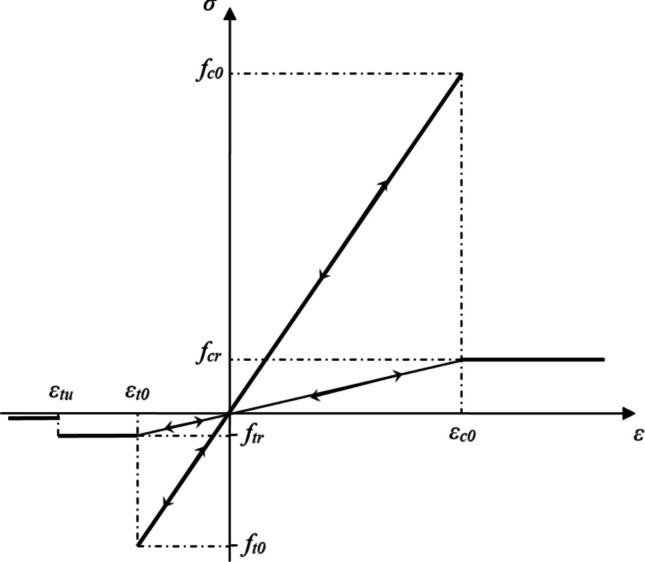
Constitutive law of an element under the uniaxial tensile and compressive stress states

Furthermore, a higher ***m*** means that the material is more homogeneous, and vice versa. In RFPA, the constitutive relation of mesoscopic elements is defined depending on the elastic damage mechanics. At the beginning of load, the constitutive curve of the elements will be linear elastic till the stress reaches the specific failure criterion. Mohr–Coulomb criterion with a tension cut-off is applied as the failure criterion, *i.e.*, the damage will occur if the stress/strain state of an element satisfies Mohr–Coulomb criterion or the maximum tensile strain/stress criterion. After that, the elastic moduli of damaged elements will degenerate with the damage degree increasing. Under uniaxial tensile or uniaxial compressive stress state (Fig. 8).

If the maximum tensile strain/stress criterion is satisfied, the damage factor $\omega$ of the damaged element under uniaxial tension as shown in Fig. 8, is expressed as (Liang 2005):

2 $$\omega =\left\{\begin{array}{cc}0& \varepsilon>{\varepsilon }_{t0}\\ 1-\frac{\lambda {{\varvec{\varepsilon}}}_{{\varvec{t}}0}}{\varepsilon }& {\varepsilon }_{tu}<\varepsilon \le {\varepsilon }_{t0}\\ 1& \varepsilon \le {\varepsilon }_{tu}\end{array}\right\}$$

where ***λ*** is the residual strength coefficient, which can be defined as ***f***_***tr***_ = ***λf***_***t0***_ = ***λE***_***0***_***ε***_***t0***_; ***f***_***t0***_ and ***f***_***tr***_ are the uniaxial tensile and residual uniaxial tensile strengths, respectively; ***ε***_***t0***_ is the threshold strain at the elastic limit; ***ε***_***tu***_ is the ultimate tensile strain of an element, describing the state when the element is completely damaged. It can be defined as ***ε***_***tu***_ = ***ηε***_***t0***_, where ***η*** is termed the ultimate strain coefficient.

Besides, Eq. (2) can be effectively extended to a complex 3D stress state from a one-dimensional stress state. Clearly, under multiaxial stress states, an element can still be damaged in the tensile mode when the equivalent major tensile strain $\overline{\varepsilon }$ reaches the threshold strain ***ε***_***t0***_. $\overline{\varepsilon }$ can be calculated as follows:

3 $$\overline{\varepsilon }=-\sqrt{{\langle -{\varepsilon }_{1}\rangle }^{2}+{\langle -{\varepsilon }_{2}\rangle }^{2}+{\langle -{\varepsilon }_{3}\rangle }^{2}}$$

where ***ε***_***1***_, ***ε***_***2***_ and ***ε***_***3***_ are three principal strains, and the angular brackets are functions defined by:

4 $$\langle x\rangle =\left\{\begin{array}{cc}x& x\ge 0\\ 0& x<0\end{array}\right\}$$

Meanwhile, the type of damages which occur when the stress state of the element researches the Mohr–Coulomb failure criterion, is termed the shear damage. Clearly, when the element is subjected to uniaxial compression and damaged according to the Mohr–Coulomb failure criterion, the expression for the damage factor ***ω*** can be described as follows:

5 $$\omega =\left\{\begin{array}{cc}0& \varepsilon <{\varepsilon }_{c0}\\ 1-\frac{\lambda {\varepsilon }_{c0}}{\varepsilon }& \varepsilon \ge {\varepsilon }_{c0}\end{array}\right\}$$

where ***λ*** represents the residual strength coefficient which is given as ***λ*** = ***f***_***cr***_/***f***_***c0***_ = ***f***_***cr***_***/E***_***0***_***ε***_***c0***_; where ***f***_***c0***_ and ***f***_***cr***_ are the uniaxial compressive strength and residual uniaxial compressive strengths, respectively; ***ε***_***c0***_ represents the compressive threshold strain.

When one element is suffering the multi-axial stress state satisfying Mohr–Coulomb failure criterion, shear damage will happen. The influence of complex stress conditions can be taken into account in this model during the damage calculation process. If Mohr–Coulomb failure criterion is triggered, the compressive threshold strain ***ε***_***c0***_ can be computed at the peak strength as follows:

6 $${\varepsilon }_{c0}=\frac{1}{{E}_{0}}\left[{f}_{c0}+\frac{1+\text{sin}\varphi }{1-\text{sin}\varphi }{\sigma }_{3}-\mu ({\sigma }_{1}+{\sigma }_{2})\right]$$

**Fig. 9:**
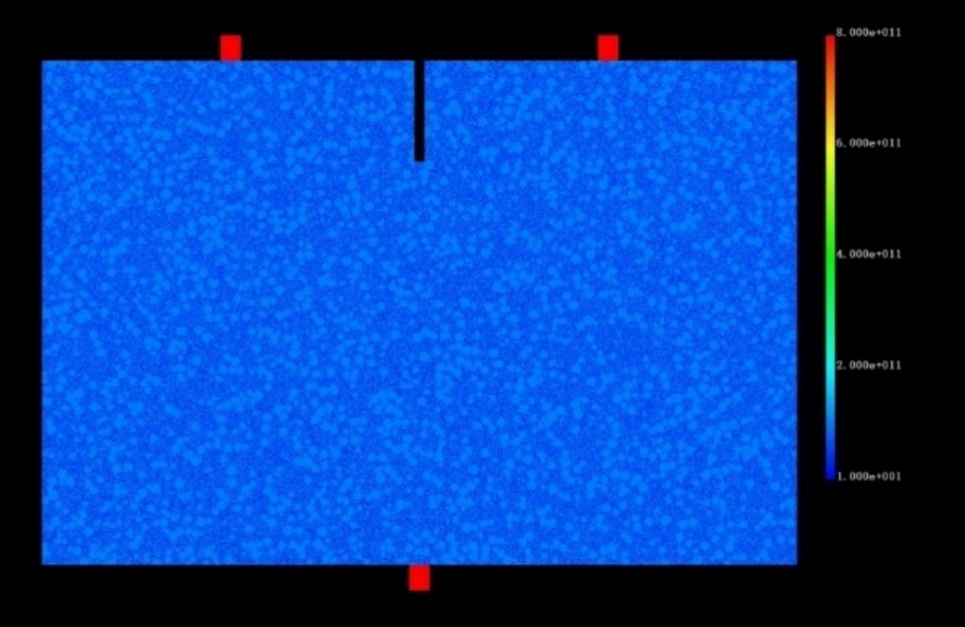
Two-dimensional mesoscopic numerical model

To investigate the mechanism of crack propagation affected by internal particles of rocks at the mesoscopic level, RFPA^2D^ is applied to build up the three-point bending model. The numerical model was composed of 594,000 mesoscopic elements and the corresponding parameters obeyed the Weibull distribution (Fig. 9). The model size was 150 mm × 100 mm with a pre-crack at the middle of the upper boundary, whose size was 2 mm (width) × 20 mm (length). The upper bearing ends were fixed along the normal direction and the lower load end was compressed with a rate of 0.001 mm/step. Numerous random particles were generated with radius changing from 0.5 mm to 1 mm. Rock matrix parameters are determined according to the references (Chen et al. 2025; Liang 2005; Feng et al. 2024) as shown in Table 1.

**Table 1:** Rock matrix parameters set

Uniaxial compression strength (MPa)	Elastic modulus (GPa)	Poisson’s ratio	Internal friction angle (°)	compression/tension ratio	residual strength coefficient	Heterogeneity coefficient
100	80	0.25	30	10	0.1	3

### Simulation results

**Fig. 10:**
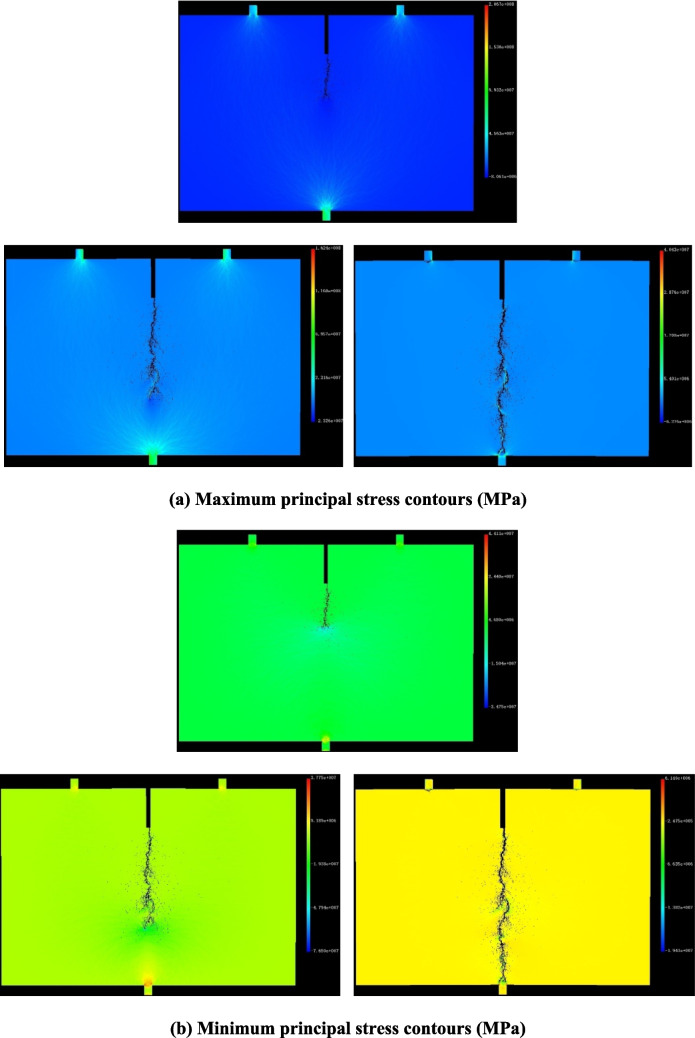
The process of crack initiation, propagation and coalescence. (**a**) Maximum principal stress contours (MPa). (**b**) Minimum principal stress contours (MPa)

The red sandstone fracturing process during the three-point bending test was simulated using RFPA^2D^. Initially, tensile stress concentrates at the tips of existing cracks, resulting in tensile failure of the rock near these tips (Fig. 10). As cracks form, the concentrated stress is released and transferred to the tips of newly formed cracks. This cycle of stress buildup, stress shadow, and stress transfer repeats, driving the continuous development of cracks. Through the initiation, propagation, and coalescence of mesoscopic cracks, a macroscopic fracture surface eventually forms, linking the pre-crack to the lower loading end.

**Fig. 11:**
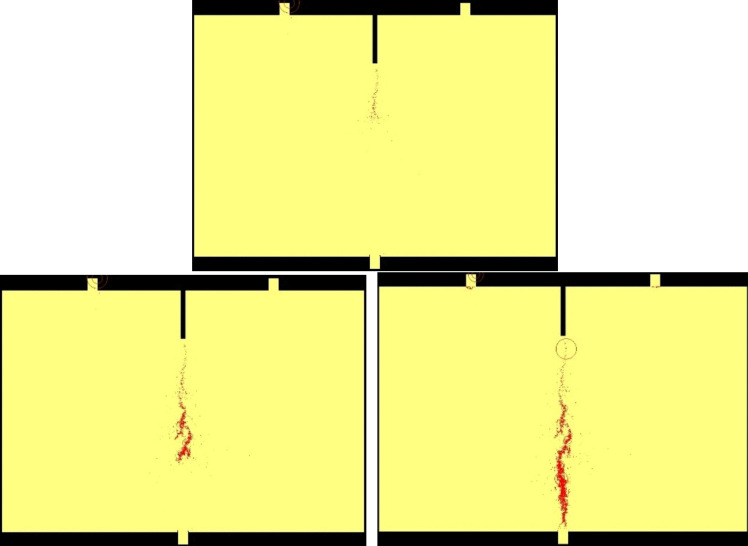
The spatial distribution of AE events during gradual fracture process

When the stress state of an element reaches the specified failure criterion, the element is considered damaged, and an AE event is represented by a ball, as shown in Fig. 11. The center of the ball corresponds to the center of the damaged element, while the radius indicates the magnitude of energy released by the AE event. The colors of the balls (blue and red) represent two types of AE events determined by the Mohr–Coulomb criterion and the maximum tensile strain/stress criterion, respectively. Figure 11 depicts the spatial distribution of AE events, clearly showing that most elements fail in the tensile mode. Initially, the energy released by failed elements near the pre-crack is relatively low. This phenomenon is also observed in the three-point bending experiment as displayed in Fig. 7b. However, as the cracks propagate downward, the magnitude of released energy increases, reflecting the more severe degree of damage, which aggress with the spatial location of AE events during the rock fracturing evolution in the three-point bending experiment as shown in Fig. 7d.

## The characteristics of fracture propagation under three-point bending

### Micro fracture morphology analysis

The red sandstone sample consisted of intermediate fine sands, with the clastic particles primarily composed of quartz (~ 50%), feldspar (~ 20%), and mica and debris (~ 30%). The microstructure of the rock sample showed the characteristics of porous cementation, and its matrix content was 5%. The size of detrital grain was 0.005**–**0.5 mm. Among them, the proportion of 0.05–0.1 mm grain was up to 40%, and the proportion of 0.1**–**0.5 mm grain was up to 60%. The shape of detrital grain was mainly presented as sub-angular shape or sub-round shape.

**Fig. 12:**
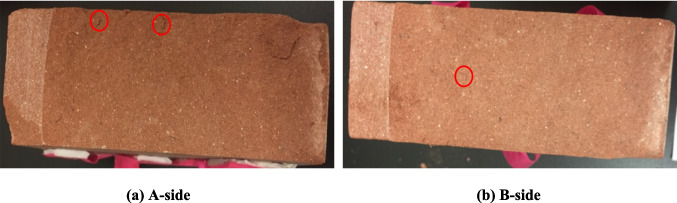
The morphology of the macroscopic failure surface. (**a**) A-side. (**b**) B-side

**Fig. 13:**
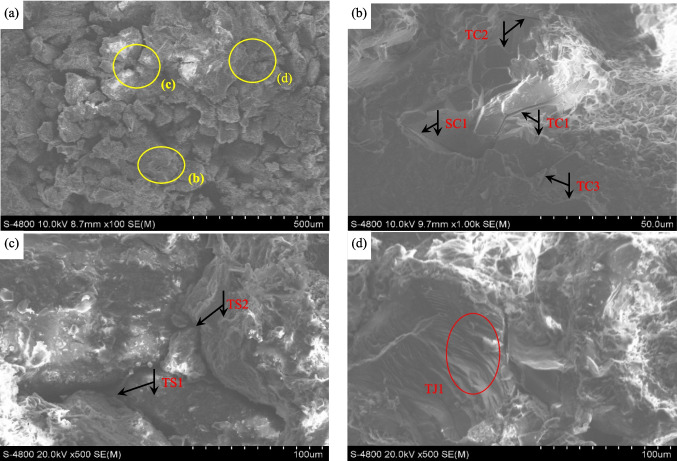
The SEM scanning results of rock detritus on the fracture surface

Rock debris at the center of the failure surface was selected to ensure that the scanned results was able to reflect the typical fracture characteristics at the micro scale. The morphology of the failure surfaces is shown in Fig. 12. Figure 13a is the scanned results of rock detritus in 100 times by SEM. Rock detritus of Area *b* is magnified 1000 times as shown in Fig. 13b and the rock detritus of Area *c* and Area *d* are magnified 500 times as shown in Fig. 13c-d.

In Fig. 13b, the fissures at Area *b* contains tensile-cracks of TC1-TC3 and shear-crack of SC1. Figure 13c displays the details of Area *c* on Fig. 13a, from which the inter-granular fracture patterns of TS1-TS2 are observed and the tensile fractures correspond to the crystalline ductile fractures. From Fig. 13d, the stepped fracture pattern of TJ1 showing the typical friction-slip fracturing surface form at Area *d* on Fig. 13a. Scanning electron microscopy (SEM) is a valuable technique for examining the micro characteristics of geo-materials. Red sandstone exhibits a granular structure, with particle boundaries weakened under stress. The morphology of rock debris was further analyzed to uncover the micro characteristics of the fracture surface. Micro-cracks consistently form at weak points, leading to fracturing along the crystal surfaces.

### Fracture propagation path affected by coarse particles

**Fig. 14:**
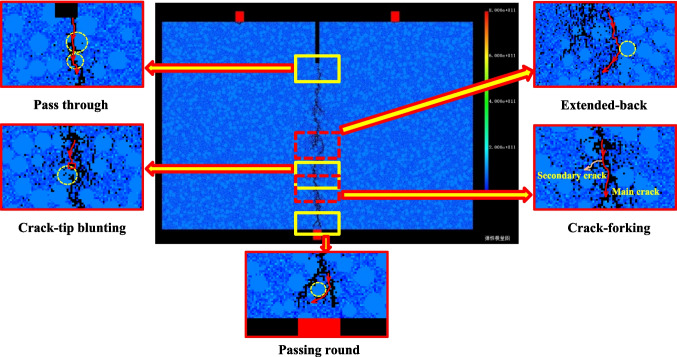
Fracture propagation modes influenced by coarse particles

As the crack propagates through the sandstone, if a small crack encounters a strong particle, the crack tip may become blunt and unable to continue growing, a phenomenon known as crack-tip blunting. When the strength of the rock matrix is lower than that of the particles, the path of the crack may change direction when it approaches a particle at a certain angle. This behavior is referred to as the extended-back mode. It influenced by the complex localized stress distribution and the material heterogeneity, a crack may branch into two cracks propagating to different directions, *i.e.,* the crack-forking mode. The bonded interface between particles and rock matrix provide the possible path for crack growth. Namely, a crack may grow around a particle, which is termed the passing-round mode. Therefore, five different modes of fracture propagation are summarized, *i.e.*, pass through, crack-tip blunting, extended-back, crack-forking and passing round, as shown in Fig. 14. For the pass-through mode, the crack separates the particles into two parts because of the significant high stress concentration when the crack reaches and penetrates into the particles.

## Discussions

**Fig. 15:**
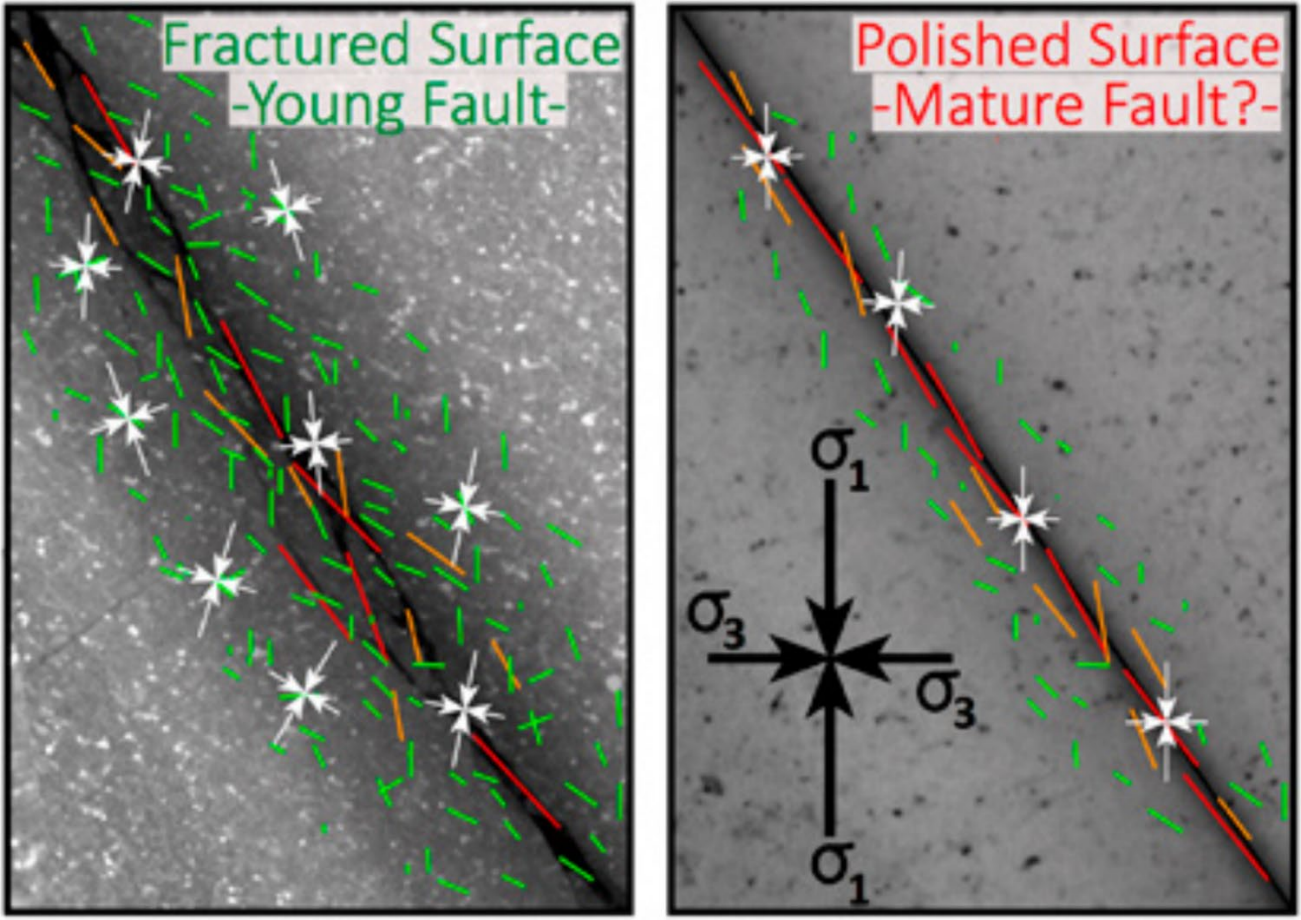
Effect of surface roughness on spatial and magnitude distributions of small cracks (green arrows) and large cracks (orange and red arrows) as well as small-scale stress field heterogeneity (white arrows) (Goebel et al. 2017)

Generally speaking, a wide spatial distribution of AE events is linked with a rough fracture surface and a high ***b*** value implies a complex focal mechanism, which could be induced by the increase of the heterogeneity of stress field at the microscopic scale. The ***b*** value can be calculated by as the slope of the log-linear relationship between the frequency of AE events and their magnitudes. Simultaneously, the localized deformation at fracture surface can promote the development of large cracks affected by the relatively uniform macroscopic stress field. This phenomenon also coincides with the findings (Goebel et al. 2017) (Fig. 15). Red sandstone specimen is mainly composed of quartz particles, feldspar particles and so on. The cementation force between particles is generally smaller than the adhesion force of particles, and fracturing behavior easily occurs at the cementation boundaries between particles. Cracks typically initiate at pre-existing structural defects, such as mineral crystal surfaces, micro-cracks, and micro-pores, when local stress concentration exceeds the fracture strength and the failure criterion is met. Generally, newly-formed local cracks exhibit a tortuous pattern due to the non-uniformity of the rock's microscopic properties and the presence of inherent defects.

**Fig. 16:**
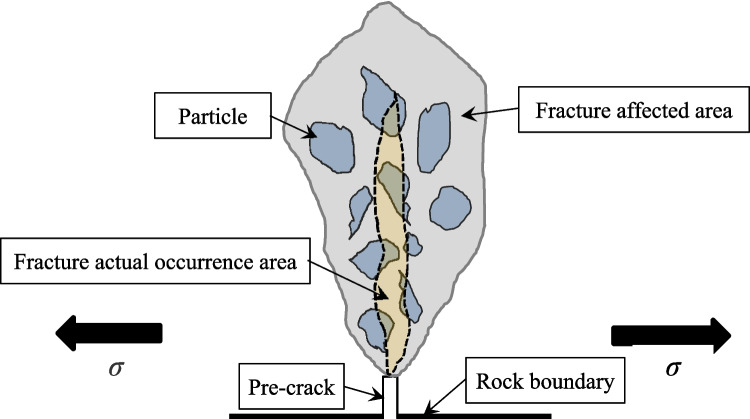
The conceptual sketch of rock crack expanding during three-point bending test

Simultaneously, Fig. 16 shows a sketch map of rock crack propagation under the Type I fracture condition. Based on the force distribution, the regions are classified into the fracture-affected area and the actual fracture-occurrence area. Cracks may initially form in the fracture-affected zone, but fracture propagation ultimately occurs in the actual fracture-occurrence area. Whether during fracture initiation or propagation, the rock will undergo varying degrees of damage, corresponding AE signals will be generated.

**Fig. 17:**
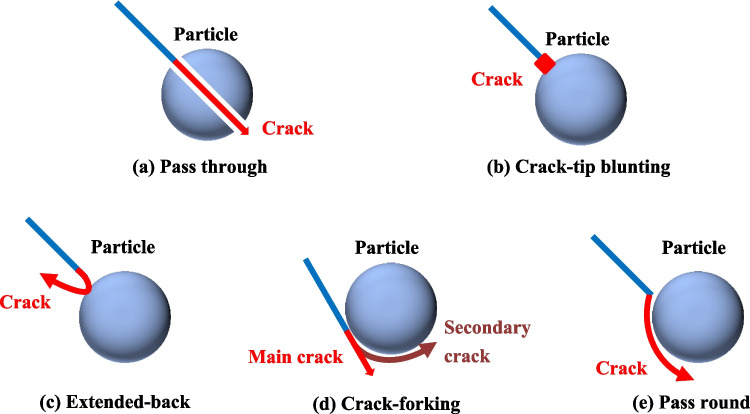
The meso-scopic crack propagation modes in red sandstones. (**a**) Pass through. (**b**) Crack-tip blunting. (**c**) Extended-back. (**d**) Crack-forking. (**e**) Pass round

The red sandstone specimen, composed of fine to medium-grained granules bound together by shale cement, exhibits a much lower bonding strength than sandy granules. As a result, it is considerably easier for fractures to occur at the cementation points between these granules. The crack propagation is further influenced by the presence of pre-existing damage defects and the geometry of the sand particles. Even if the force grows up linear and the direction of the force is constant, cracks mainly propagate along the cement surfaces and present a variety of crack propagation modes including pass through, crack-tip blunting, extended-back, crack-forking, passing round, etc*.*, as shown in Fig. 17.

**Fig. 18:**
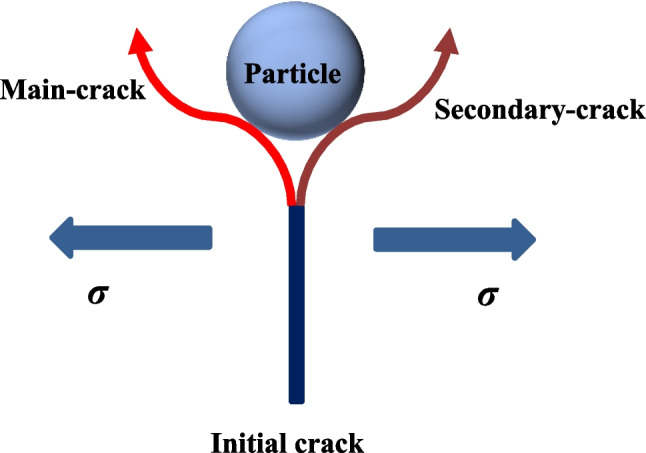
Sketch map of secondary cracks during tensile crack propagation in the presence of silt particle

Additionally, the observed tensile fracture surface is conceptualized in Fig. 18. Actually, a small number of fine sandstone particles are suspended on the fracture surface, and the fracture surface has a certain roughness. This is because of the fact that some sandstone particles are relatively small, and under the high tensile stress, the initial crack will occur along the main-crack orientation or secondary-crack orientation of the sandy particles.

## Conclusions

1. The gradual energy release during the fracturing of red sandstone under the three-point bending test can be categorized into four distinct stages. The predominant crack type observed in red sandstone fracturing is tensile, with a small proportion of shear-type cracks. The roughness of the fracture surfaces was minimal, and the fracture direction was nearly vertical.
2. The instability precursors of red sandstone specimen fracturing under the three-point bending test exhibit distinct features across IR, AE, and crack types. Infrared thermography reveals the scattering of significant amounts of high-temperature debris. AE analysis shows that cumulative energy reaches its peak value, with AE events concentrated at the bearing ends and the loading side. Tensile cracks dominate, while shear cracks account for only a small percentage.
3. During the fracturing of red sandstone under the three-point bending experiment and numerical simulations, distinct behaviors were observed depending on the level of tensile stress. At low tensile stress levels, a macro tensile crack was observed in the center of the rock specimen. The resulting macroscopic fracture morphology exhibited typical brittle failure characteristics. At higher tensile stress levels, the granular structure of the rock specimen became increasingly weakened, leading to the formation of micro-cracks along the weaknesses of crystal surfaces.
4. The repeated processes of stress buildup, stress shadow, and stress transfer played a critical role in driving the continuous development of micro-cracks. Five distinct modes of fracture propagation were identified: pass-through, crack-tip blunting, extended-back, crack-forking, and passing around.

## Data Availability

The data underpinning this publication can be accessed from Brunel University of London's data repository (Brunelfigshare) here under a CCBY license (with the DOI of 10.17633/rd.brunel.28540166).
